# Conspecific brood parasitism in the tropics: an experimental investigation of host responses in common moorhens and American purple gallinules

**DOI:** 10.1002/ece3.26

**Published:** 2011-11

**Authors:** Susan B McRae

**Affiliations:** 1 Department of Biology, East Carolina UniversityGreenville, North Carolina; 2 Smithsonian Tropical Research InstituteApartado Postal 0843-03092, Panamá, República de Panamá

**Keywords:** Breeding synchrony, egg rejection, *Gallinula chloropus*, intraspecific brood parasitism, *Porphyrula martinica*, Rallidae, seasonality, neotropics, wetlands

## Abstract

Species occupying a broad latitudinal range may show greater phenotypic plasticity in behavior than species with smaller ranges or more specific habitat requirements. This study investigates for the first time the occurrence of conspecific brood parasitism (CBP) in sympatric tropical populations of the common moorhen (*Gallinula chloropus pauxilla* Bangs) and the American purple gallinule (*Porphyrula martinica* L.). CBP occurred in at least 20% (*N* = 76) of common moorhen nests on the Rio Chagres in Panama. Half (*N* = 20) of the parasitic eggs were accepted, but 10 were destroyed or ejected from host nests. Introductions of experimental eggs into nests revealed hosts were more likely to accept parasitism later in the host's laying period and during incubation, consistent with expectation of an adaptive response. CBP was not detected in a small sympatric population of American purple gallinules. Members of this population did not eject experimental eggs, suggesting a lack of experience with costly CBP. Contrasting ecological factors help explain why these two species of rail (Family Rallidae) differ in regard to CBP. Purple gallinule territories were sparse, owing to the distribution of preferred habitat. Moorhens flocked outside of the breeding season. They nested more synchronously, at higher densities, and primarily in ephemeral floating vegetation. Further, moorhens suffered a rate of nest loss nearly double that of American purple gallinules, and this increased over the course of the breeding season. Moorhen clutches were larger on average, and more variable in size than those of purple gallinules. Reproductive effort and rate (seasonality) constitute important life history differences between these species that may constrain the evolution of reproductive tactics. Comparing these sympatric populations, and others differing in life-history traits and ecological constraints, highlights the role of risk management in the evolution of CBP.

## Introduction

Avian brood parasitism remains an enduring model of coevolution between hosts and parasites (Payne 1977; Rothstein and Robinson 1998; Davies 2000). Conspecific brood parasitism (CBP) occurs when a female lays her egg(s) in the nest of conspecific hosts, leaving them responsible for their care. This flexible reproductive tactic has been described in numerous taxa including 200–300 species of birds (Davies 2000; Yom–Tov 2001). The unique interplay between hosts and parasites derived from the same population has led to the recognition of a role for CBP in shaping life-history trait evolution and population dynamics (Eadie et al. 1998; Lyon and Eadie 2008).

Studies of brood parasitism commonly incorporate experimental assessment of host responses. The experimental introduction of model eggs can be used to determine more accurately the extent to which parasitism occurs in a population where, for example, host rejection behavior may lead to underestimates of parasitic egg numbers (Rothstein and Robinson 1998). Where natural brood parasitism is not detected, finding sophisticated host responses to foreign eggs could indicate that a population has had a history of brood parasitism (Lindholm and Thomas 2000; Lahti 2005).

In a conspecific host nest, a parasitic egg's fate is in part determined by the timing of parasitic laying (McRae 1995). It may also vary according to the relationship of the host to the parasite (McRae and Burke 1996; Andersson 2001; Lopez–Sepulcre and Kokko 2002; Roy Nielsen et al. 2006b). Conspecific host responses range from nest desertion to raising the offspring as its own. Another possibility is for the host to recognize and reject specifically the parasitic egg. The problem of egg recognition in birds has received much attention but many questions remain about the mechanism of discrimination and the effects of learning and experience on this process (Rothstein 1975; Lotem et al. 1995; Lyon 2007; Shizuka and Lyon 2010).

Variation in behavior resulting from ecological differences among populations may be expected to be most extreme where the species occurs over a broad latitudinal range. The common moorhen *Gallinula chloropus* has a cosmopolitan distribution covering five continents spanning latitudes from 50°N to 40°S of the equator (Taylor and van Perlo 1998). Common moorhens are among a number of rail species that have been found to exhibit CBP, and the behavior has been studied in detail in temperate populations (Gibbons 1986; Ueda et al. 1993; McRae 1995, 1997; Post and Seals 2000). Detailed study of the tactics of parasitic and nonparasitic female moorhens has shown that females employ parasitism both as a means of enhancing their seasonal reproductive success when they have abundant resources, and alternatively as a salvage strategy or when nesting opportunities are compromised (McRae 1998). Each of these alternatives predicts a different pattern to the distribution and timing of nest parasitism in the population. The success rate of individual brood parasites is particularly sensitive to the timing of parasitism in relation to the host nest cycle, an important determinant of survival for parasitic chicks (McRae 1995).

American purple gallinules nest in the shallow margins of lakes, rivers, and marshes from Florida to subtropical South America. They lead a more terrestrial lifestyle than common moorhens (Taylor and van Perlo 1998). They rarely swim and prefer to nest in dense emergent vegetation on the shoreline. Territories are defended by monogamous pairs (Helm 1994). Juveniles commonly remain on the natal territory and help to rear siblings from subsequent broods (Hunter 1985).

Empirical data detailing spatial and temporal distribution of CBP in populations in different environments are scarce (Lyon and Eadie 2008). Studies of reproductive strategies of birds in tropical populations are underrepresented, but may offer insights into how particular ecological factors affect behavior (Macedo et al. 2008). Tropical habitats typically support a long breeding period with little synchrony in nesting. Synchrony is believed to influence rates of reproductive interference by varying the degree of opportunity for such behavior to occur (Stutchbury and Morton 2001).

In a year of exceptionally high nest predation, CBP rate in a common moorhen population in Britain increased dramatically (McRae 1997). CBP rates among nest sites of goldeneye ducks (*Bucephala clangula*) have also been found to increase in relation to increased risk of nest predation (Pöysä 1999). Avian nest predation rates are typically high in tropical systems (Robinson et al. 2000). Thus, tropical populations may be expected to exhibit higher rates of CBP.

This is the first study to document CBP in rails in the neotropics. My first objective is to describe the rates of occurrence of CBP in two sympatric tropical populations: a tropical subspecies of the common moorhen, *G. c. pauxilla* (Bangs), and the American purple gallinule. Second, I investigate experimentally whether hosts in these tropical populations have evolved defenses against brood parasites. Finally, I document seasonal patterns in nest initiation, nest success and predation rates in both species, and compare ecological factors and life-history traits that may account for variation in behavior between the species.

## Materials and methods

### Study site

**Figure 1:**
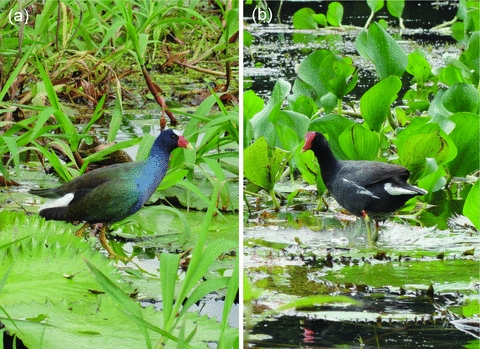
(A) American purple gallinule and (B) neotropical common moorhen on the Rio Chagres, near Gamboa, Panama.

Breeding common moorhens and purple gallinules were observed at four sites on the Rio Chagres, near the village of Gamboa, Republic of Panama (Fig. 1). A survey of study sites around the village of Gamboa during June–July 1998 and in January 1999, revealed that these locations, all within 3 km of where the Rio Chagres feeds into the Panama Canal, had the highest densities of rails in the region. For additional details of the study area see Emlen and Wrege (2004).

**Figure 2:**
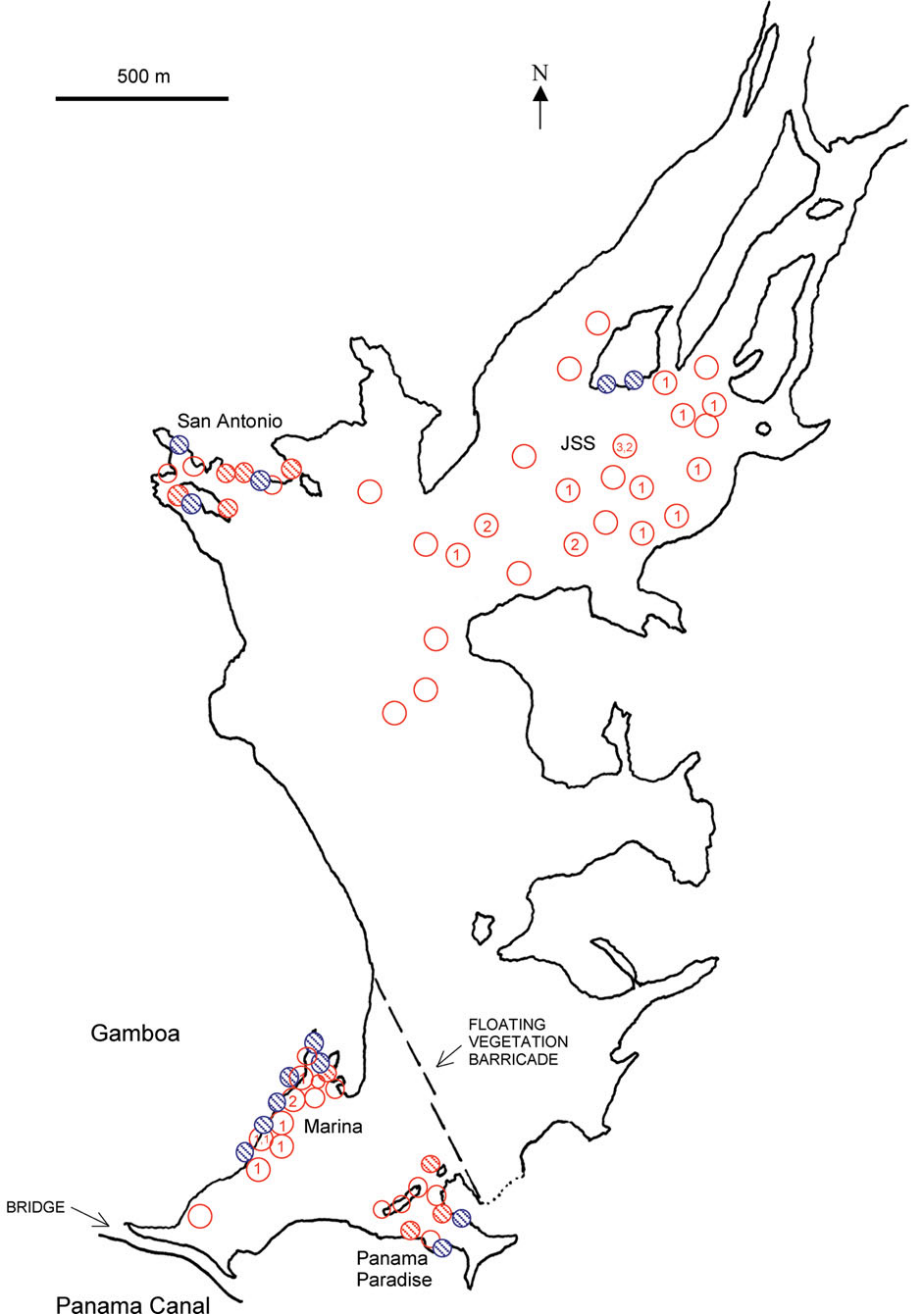
Distribution of territories of common moorhens (red) and American purple gallinules (blue) on the Rio Chagres near Gamboa, Panama. Open circles represent impermanent breeding territories. Hatching indicates “resident” territories that were already established at the start of the study. Circle sizes are not to scale. Parasitic eggs were only found in impermanent territories of moorhens. The number of eggs laid by brood parasites on those territories is indicated. Parasitic eggs laid in two bouts are separated by a comma. All parasitic eggs were laid in nests on impermanent territories at the two sites with the greatest numbers of nesting moorhens.

Common moorhen territories were aggregated in four distinct regions along the river (Fig. 2). In two of these areas, “San Antonio” (9°07′50″N, 79°41′24″ W) and “Panama Paradise” (9°06′45″N, 79°41′24″ W), pairs were found defending shoreline territories from the beginning of the dry season (in this region of Panama, December–April). In two others, “Jacana Study Site” (hereafter, “JSS”) (9°07′48″N, 79°41′6″ W) and “Marina” (9°06′54″N, 79°41′30″ W), pairs emerged from two foraging flocks between early February and mid-April, and began to defend territories in the ephemeral floating vegetation that accumulated throughout the dry season. Most of the moorhen population had paired off and settled on territories by late March. By contrast, purple gallinules were found at low-density defending territories on the margins of the river continuously throughout the study period. Both species were studied intensively between 1 February and 15 July, 1999. Sites continued to be surveyed for breeding activity at least monthly through December 1999.

Nests were found before laying began or early in the laying period by systematically searching aquatic vegetation in territories defended by breeding pairs. Nests were checked daily between 07:00 and 16:00 hours CST during the laying period, and at least once every 2 days during the incubation period. Each egg was marked individually with indelible ink on the day it was found. Parasitism was inferred when (1) more than one egg appeared within a 24-hour period, or (2) an egg was laid 2 or more days after the host had stopped laying. DNA fingerprinting has confirmed these field techniques for identifying parasitic eggs are reliable (McRae and Burke 1996). Nests were followed daily throughout the incubation period, and the fate of each egg was documented for both species. Disappearances of marked eggs were noted during daily nest checks, and the nests were searched for possible buried eggs. Eggs remaining after incubation ended were collected and checked for fertility.

### Experimental egg introductions

Experimental egg models were cast with plaster of Paris, and painted with acrylic paint (base color cream for moorhens, pale pink for American purple gallinules). Spots were added with an indelible brown felt marker to resemble natural eggs. Dimensions of these model eggs were within the range observed for eggs in these populations. When available, I also used freshly laid eggs obtained from deserted nests of conspecifics. In total, eight real moorhen eggs and two real purple gallinule eggs were used. No differences in response by experimental hosts were found between real and model eggs (see results).

For each experiment, I selected an experimental egg that looked different from the host eggs to facilitate discrimination by the hosts. Behavioral observations suggested that members of this moorhen population lay close to dusk. Therefore, model eggs were introduced in experimental nests between 17:30 and 18:30 hours CST. Experimental introductions were conducted at one of the following times: “Day 0” (before the host had begun to lay), Day 1 (after the host had laid one egg), Day 2 and Day 4 of the host's laying period, and after host clutch completion. These days were selected because differences in host response rates were observed in this time interval in a previous study of a temperate population of moorhens (McRae 1995). To control for potential disturbance of visiting experimental nests twice in one day, seven additional nests were visited at the same time in the late afternoon on Day 1. In each case, a hand was reached in the nest without adding an experimental egg.

I conducted similar experiments at a smaller number of nests of the sympatric American purple gallinule. It is not known at what time of day the purple gallinules lay, but observations of the shell surface appearance of recently laid eggs in conjunction with sequence data suggested a variable daytime hour. Model eggs were therefore added between 06:50 and 12:30 hours CST in an attempt to emulate conspecific laying behavior as closely as possible.

Parasitic and experimental eggs were photographed with the hosts’ eggs within 24 hours of their introduction in the nest. All complete clutches were also photographed with the eggs laid out in the sequence they were laid. An experimental egg was considered “Accepted” if it remained in the nest and was incubated up to the point that the first host egg hatched. The plaster model eggs incurred damage when pecked or gnawed, enabling me to distinguish between host responses of egg destruction and nest desertion versus predation. Experimental nests that were depredated during incubation were excluded from the analysis.

All procedures were reviewed and approved by animal care and use committees of the Smithsonian Tropical Research Institute and the Autoridad National del Ambiente, Republic of Panama.

## Results

### Tropical common moorhens on the Rio Chagres, Panama

The tropical common moorhen population on the Rio Chagres study site consisted of at least 70 pairs and additional floaters. In January, the second month of the dry season, 10 pairs already occupied territories at the “San Antonio” and “Panama Paradise” sites (hereafter, “resident territories”). Based on a survey on March 31, the majority of common moorhens (81/105) were in two flocks near the Marina/Panama Paradise and JSS study sites, respectively. As the river level receded, pairs emerged from these flocks and began defending territories surrounding islands of floating vegetation (hereafter “impermanent territories”). Floaters of unknown sex were observed at the JSS study site throughout the breeding season. The moorhens nested predominantly in floating hyacinth *Eichhornia crassipes* (an invasive species) and water lettuce *Pistia stratioles*, and also in emergent reedy vegetation at the shoreline (*Scleria eggersiana* and *Typha domingensis*). Laying began on 3 March, and continued through 6 July, 1999. Fifty-four territories were monitored on a daily basis at four sites on the river.

### Nesting by tropical moorhens

**Figure 3:**
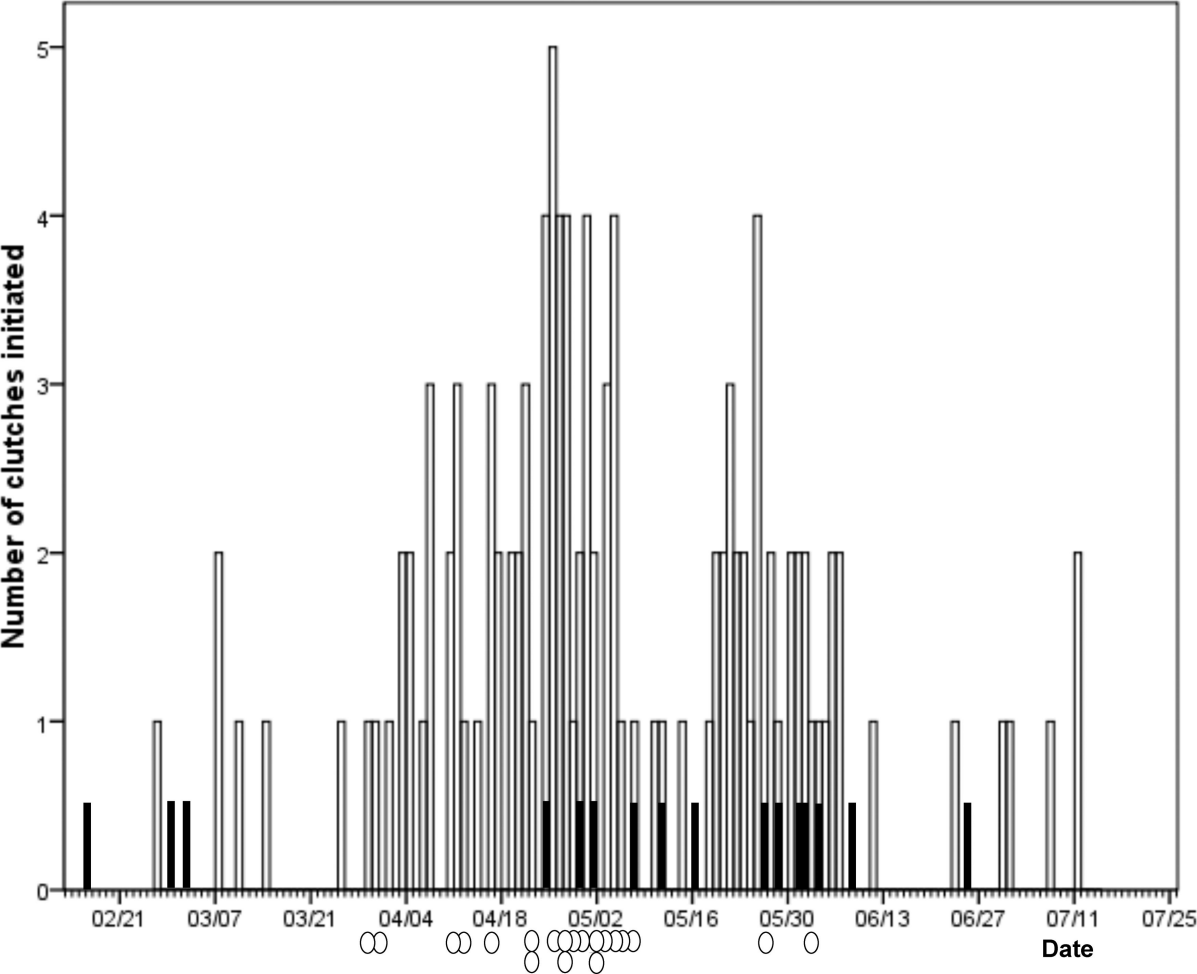
Temporal distribution of nest initiation dates of common moorhens and purple gallinules on the Rio Chagres. The number of moorhen clutches initiated each day is indicated by open bars. Each egg symbol below the date scale represents the start date of a parasitic laying bout by a common moorhen. The peak of parasitic laying coincided with the peak in clutch initiation. No more than one purple gallinule nest was initiated on any given date. These dates are indicated by the short black bars. There were no parasitic purple gallinule eggs.

I monitored 115 active nests of which 76 were found before laying began or within the first 2 days of laying (between 7 March and 7 June, 71% of 103 nests were found by Day 2). Peak nest initiation occurred in late April (Fig. 3). Of the 115 nests, only 34 (30%) survived to hatch. Predation accounted for 42% of nest losses (14 nests during the laying period, 21 during incubation, 13 during hatching). A variety of predators were believed to be responsible including aquatic birds such as herons (Ardeidae), caymen (*Caiman crocodylus*), and possibly snakes. Other sources of nest loss were: flooding (*N* = 6) and nest desertion (*N* = 18). Several nest desertions occurred in the early stages of laying, including three in response to experimental parasitism and at least four due to natural parasitism. Other desertions occurred toward the end of the breeding season when nests built on hyacinth islands floated down the river after the water level rose several meters.

Nest success was significantly better for moorhens on resident territories (nine [56%] of 16 nests hatched) compared with those on impermanent territories, many of which were on ephemeral islands of floating vegetation (25 [26%] of 95 nests hatched; χ^2^ = 5.92, df = 1, *P* = 0.02). Two entirely infertile clutches laid in one resident territory and two destroyed experimental nests were excluded from this analysis.

### Brood parasitism and host responses among tropical common moorhens

Most parasitism occurred at the peak of laying (Fig. 3). Of 76 nests with complete laying histories, 15 (20%) were parasitized by conspecifics as confirmed by laying sequence data. If all nests where at least one egg was laid are considered, the estimated rate of parasitism was 20 of 115 nests (17%). All parasitized nests were in impermanent territories (Fig. 2); none of the 17 nests belonging to moorhens on resident territories was parasitized (χ^2^ = 4.89, df = 1, *P* = 0.03). Parasitized nests were in ephemeral floating vegetation, except for three that were in shoreline vegetation.

**Figure 4:**
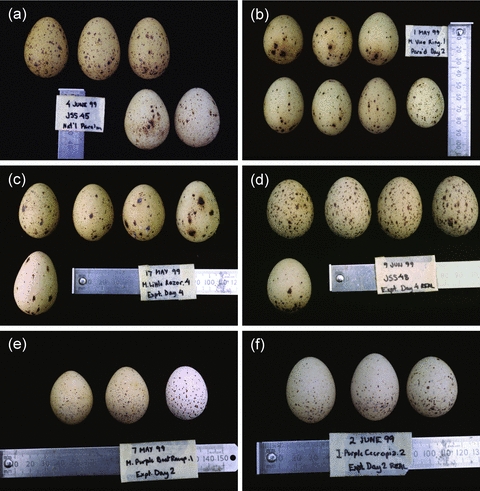
Eggs from selected parasitized and experimental nests. In each photograph, host eggs are shown along with the parasitic or experimental model eggs. Eggs from two parasitized tropical moorhen nests are displayed: (A) shows three host eggs with two parasitic eggs (laid on Days 3–4) in the bottom row, and (B) shows six host eggs with a single parasitic egg (laid on Day 2) at bottom right. Partial tropical moorhen clutches photographed following “Day 4” experiments using (C) a plaster model egg (lower left) and (D) a real moorhen egg (lower left). Partial American purple gallinule clutches photographed following “Day 2” experiments using (E) a plaster model (far right) and (F) a real purple gallinule egg (far right).

Eggs of individual females were visually distinguishable based on size, color, shape, and spot pattern (Fig. 4). The weights and dimensions of tropical moorhen (*G. c. pauxilla)* eggs were similar those of *G. c. chloropus* eggs in a European population (McRae 1995, Appendix S1). Twenty (4.6%) of the 434 eggs found in moorhen nests were parasitic. This is a conservative estimate, considering only nests with good sequence data. Ten (50%) of the parasitic eggs went missing from the nest and were presumed destroyed or ejected. By contrast, only eight of 414 host eggs went missing (χ^2^ = 99. 1, df = 1, *P* < 0.0001).

Host responses varied in relation to the timing of parasitism (Table 1A). Three parasitic eggs laid before the hosts began laying were identified by matching distinctive egg markings to a neighboring hen or excluding the host after she laid. These were found destroyed.

**Table 1:** Tropical common moorhen host responses

(A) Host responses to natural parasitic eggs. Each number under “Host response” denotes the number of parasitic eggs laid in a host nest. Five instances where putatively parasitic egg(s) were laid in empty nests are indicated in brackets (see text).					

		Host response			
		
Timing of parasitism	*N*	Destroyed	Deserted	Ejected	Accepted
Before host laid	4 (5)	1,1,1,1, (2)	(1,1,1,1)		
Day 1	3		1,1	3	
Day 2–5	4			2,1^1^	1, 2^2^
Incubation period	5				1^1^,1,1,1,2

**(B) Host responses to experimental parasitism. Responses are defined below.**					
		**Host response**			
		
Timing of Experiment	*N*	Destroyed	Deserted	Rejected	Accepted
Before host laid	5	5 (100%)	0	0	0
Day 1	13	0	3 (23%)	2 (15%)	8 (46%)
Day 2	10	0	0	2 (20%)	8 (80%)
Day 4	10	0	0	1 (10%)	9 (90%)
Incubation	6	0	0	1 (17%)	5 (83%)

1 same nest.

2 host may have ejected one of own eggs.

Destroyed = egg found broken in nest, or removed and the nest unused.

Deserted = egg left cold but undamaged.

Rejected = egg ejected from or buried in an active nest.

Accepted = egg retained in nest and incubated.

Two host pairs deserted their nests within 18 hours of being parasitized the day their first egg was laid. At another nest, three eggs laid parasitically on host laying Days 1–3 were ejected. At four host nests, one or two parasitic eggs were laid between Days 2 and 5; two hosts ejected the parasitic eggs and two hosts accepted. In one of these nests (eggs pictured in Fig. 4A), a second parasitic egg appeared but no host egg was found the next day, raising the possibility the hosts may have ejected their own egg. Five host pairs, one of which ejected an early parasitic egg, accepted one or two parasitic eggs laid after host clutch completion (Table 1B).

Parasitic moorhen eggs fared poorly. Among nine parasitic eggs in seven nests that were accepted by the hosts, only one survived to hatch. Predation (four nests) and insufficient incubation due to being laid after host clutch completion were the causes.

### Experimental parasitism in moorhens

The temporal pattern of responses of moorhen hosts to experimental parasitism paralleled that of host responses to natural parasitic eggs. For experimental introductions before the host laid, and on host laying Day 1, I used only realistically painted plaster model eggs. For experiments on Day 2 or later, some freshly laid moorhen eggs were used (Fig. 4). Experimental host responses did not differ significantly based on experimental egg type used (Acceptance rates: Day 2 expt., 4/6 plaster models, 4/5 real eggs, Fisher's exact *P* = 0.99; Day 4 expt., 8/8 plaster models, 1/2 real eggs, Fisher's exact *P* = 0.20; after clutch completion expt., 4/5 plaster models, 1/1 real eggs, Fisher's exact *P* = 0.99). Therefore, results from experiments using real and plaster models were pooled (Table 1B).

Of five eggs placed in nests before the host began laying, three were destroyed (showing peck marks and partial burial), and two removed within 24 hours and the nest unused. Acceptance of parasitic eggs increased the later the eggs were added to host nests (Table 1B). In both cases where hosts ejected the egg placed in the nest on Day 1, as well as in the case of an experimental egg added on Day 4, egg rejection occurred between 13 and 40 hours after the experimental introduction. In one case of a model egg added on Day 2, the experimental egg and a host egg both disappeared the same day, within 54 hours of the experimental introduction. Two other Day 2 experiments resulted in the model egg being rejected during incubation, one by burial, the other by ejection. Six of 34 experimental eggs introduced during the host laying period went missing and were considered rejected. By comparison, a significantly lower proportion of host eggs went missing during laying or incubation (8/414; χ^2^ = 28.5, df = 1, *P* < 0.0001). Only three of the eight “host” eggs that went missing were from unparasitized, nonexperimental nests. Thus, mistaken or accidental egg rejection may have occurred.

One of six eggs introduced during the host's incubation period was rejected (Table 1B). This egg was introduced 2 days after clutch completion, and ejection occurred between 4 and 5 days later. The other experimental eggs were introduced 3, 4, 7, 8, or 18 days after clutch completion, and all were accepted.

Anecdotally, a single additional Day 1 introduction of a model egg painted red resulted in the hosts deserting their nest within 18 hours. Two days later, the host egg was found pecked but not eaten, and the model was partly buried in the nest. Further trials were not possible due to limited numbers of nests.

In six of seven control nests that were visited and a hand placed in the nest during the same time interval in which experiments were carried out, the host laid before the next morning, and other aspects of nesting were not different from nests visited only once per day. The seventh control nest had to be excluded because it was on a hyacinth island that floated 30 m away overnight. It was found the next day with its single marked egg pecked open but uneaten. Overall, host egg rejection responses were unlikely to have been caused by investigator disturbance.

### American purple gallinules on the Rio Chagres, Panama

Thirteen pairs of American purple gallinules defended territories in emergent vegetation along the shore. Their patchy distribution coincided with shallow river margins that support suitably dense stands of the thick, reedy vegetation (*S. eggersiana* and *T. domingensis*) preferred for nesting. Prior to the focal study period, four-times weekly visits to the Rio Chagres during the rainy season (in June–July 1998) revealed that while most of the common moorhens had returned to flocks, American purple gallinules were still defending territories. At least two females laid in mid-June 1998, and at least five of the 13 pairs on territories had juveniles (1–2 months old as estimated from plumage). A census on February 6, 1999 found nine further pairs of territorial purple gallinules (some with juveniles) far upstream from the JSS site (Fig. 2).

### Lack of brood parasitism in American purple gallinule nests

Fewer American purple gallinules bred in the study area, so sample sizes were small. No parasitic eggs were found in any of the 14 nests monitored in 1999, nor in two nests monitored in 1998. Purple gallinule eggs laid by different females were distinguishable by size, shape, and color. They were pale pink with small brown and mauve spots, but were less pigmented than moorhen eggs. For mean dimensions and weights of American purple gallinule eggs, see Appendix S1.

American purple gallinules laid on average one egg per day. However, among nine of 12 complete clutches monitored daily, an egg in the sequence was missed. This occurred on the third (*N* = 1), fourth (*N* = 7), or fifth (*N* = 1) day of the laying sequence. Individual nests were checked at approximately the same time each day. The pattern of laying suggests purple gallinule hens lay in daylight, but at intervals greater than 24 hours.

### Experimental parasitism in purple gallinules

Introductions of experimental eggs were conducted in the same manner as those with moorhens on host laying Days 1, 2, 4, or after clutch completion. Nine of 11 experimental eggs were made of plaster of Paris covered with acrylic paint. The remaining experiments, on Day 2 and after clutch completion, respectively, used real eggs from partially depredated nests. These were both accepted by hosts as were seven of the nine plaster eggs.

In five of the six experiments in which a model egg was introduced after the host purple gallinule had laid one egg (Day 1), the egg was accepted and incubated. In the exceptional case, the nest was deserted within 24 hours, and the female laid immediately (without missing an egg in the laying sequence) in another nest on the territory 4 m away. A control experiment in which the experimenter visited a nest for a second time on Day 1 and placed a hand in the nest produced no response: a host egg was laid and the nest incubated normally.

Only two Day 2 and two Day 4 experimental trials were performed. In each case, the model egg was accepted and incubated by the hosts. In one Day 4 experiment, the model egg was buried in the nest 18 days after introduction. I did not consider this to be a case of egg rejection since it occurred after the host eggs had begun hatching. In a single trial conducted after clutch completion, the experimental egg was accepted and incubated by the hosts.

### Interspecific interactions

Common moorhen territories were more abundant and widely distributed during the period of intensive study. Consequently, purple gallinule nests were always closer to moorhen nests than to conspecific nests (Mean conspecific nearest neighbor = 309 ± 116 m, Mean nearest moorhen nest = 32 ± 9 m, *N* = 10; Paired sign test, *P* = 0.008). In spite of their smaller size (Taylor and van Perlo 1998), purple gallinules vigorously defended their territories against common moorhens. No interspecific brood parasitism was detected.

Several moorhen territories encroached on those of purple gallinules (Fig. 2). Purple gallinules were believed responsible for destroying seven (6% of 115) moorhen clutches, representing 20% of the 35 moorhen nests that were depredated during laying and incubation. I found no evidence that moorhens damaged purple gallinule nests or eggs.

### Clutch size, nest success, and nesting synchrony

Considering only complete clutches, I found no significant difference in host clutch size among moorhen nests parasitized with one or two eggs versus nonparasitized nests (analysis of variance [ANOVA] *F*_2,65_ = 0.15, *P* = 0.86). Nor was there a difference in the number of host eggs laid in nests where an experimental egg model was accepted (mean ± SE = 6.1 ± 0.3) versus unmanipulated nests (mean ± SE = 5.8 ± 0.2; ANOVA *F*_1,76_ = 0.82, *P* = 0.37). Therefore, clutch size data were pooled for all moorhen nests.

**Figure 5:**
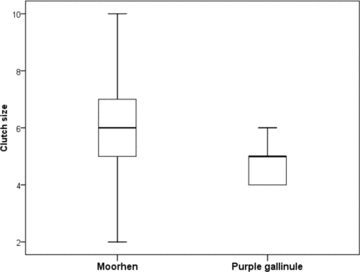
Clutch sizes of tropical moorhens (*N* = 81) had a significantly higher mean and variance than those of purple gallinules (*N* = 11). Box plots show means, 5th, 25th, 75^th^, and 95th percentiles.

Clutch size varied more widely among nests of the moorhens (range = 2–10, *N* = 81) than among purple gallinule nests (range = 4–6, *N* = 11; Fig. 5). Mean clutch size of the tropical moorhen (5.7 ± 0.2, *N* = 81) was significantly greater than that of the sympatric purple gallinule (4.6 ± 0.2, *N* = 11; *t* = 4.23, df = 26, *P* < 0.001).

An important difference between these sympatric populations was the rate of nest success. Only 30% of 115 moorhen nests initiated survived to hatch. By contrast, 77% of 13 purple gallinule nests followed through incubation hatched (χ^2^ = 11.07, df = 1, *P* = 0.0009).

**Figure 6:**
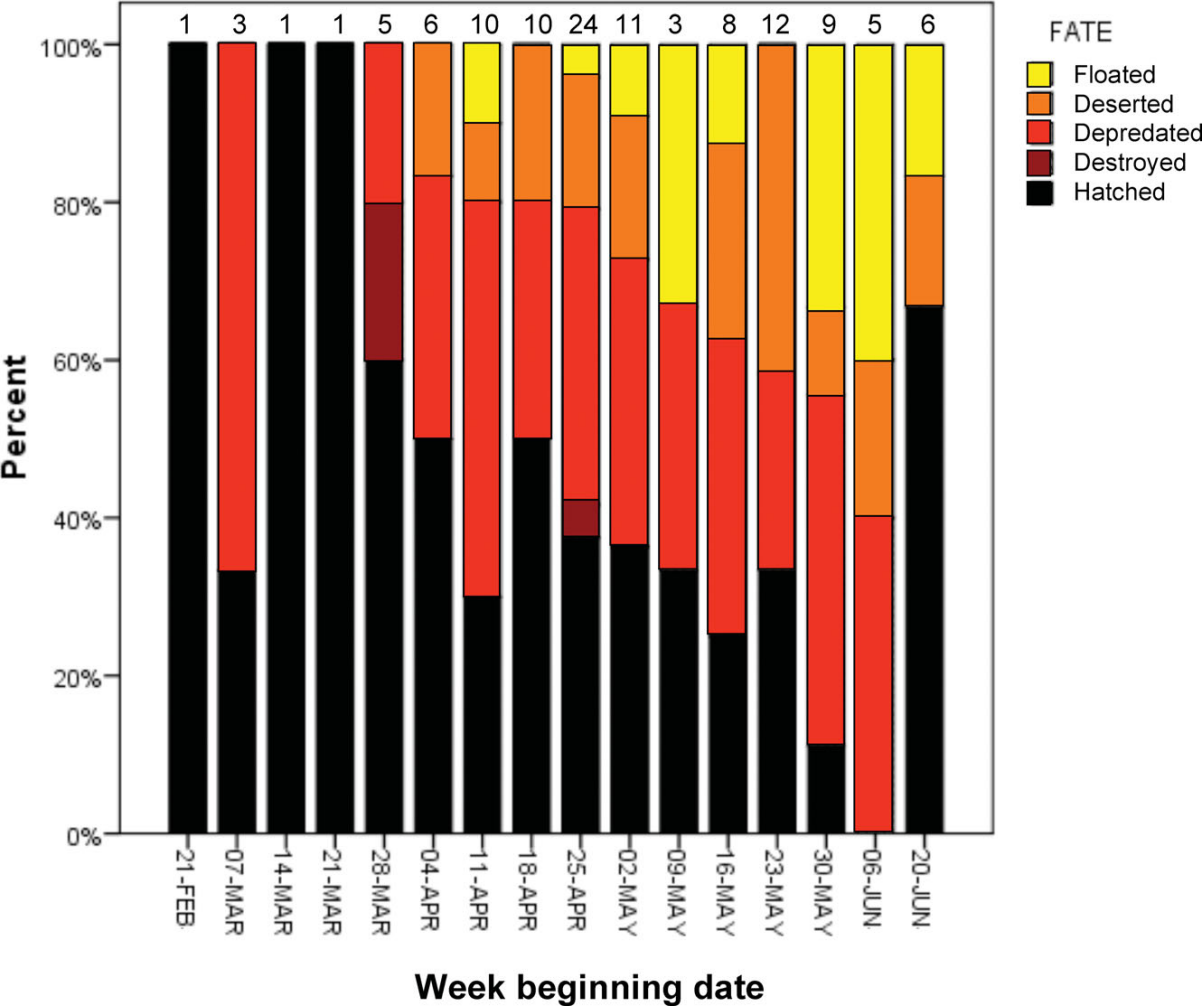
The proportion of moorhen nests surviving to hatch declined over the season (χ^2^_1,15_ = 69.90, *P* < 0.001). Fates of moorhen nests over the duration of study are shown by week of clutch initiation. Each bar illustrates the percent of nests that survived to hatch, were depredated at the egg stage, were destroyed after parasitism or deserted, or whose island substrate floated off down the river. Those categorized as “Floated” ended up far downstream (more than 1 km in some cases) and were inevitably abandoned. Other causes of desertion varied and included responses to parasitism and other disturbances such as break up of the floating island vegetation. The date indicated is the beginning of each week-long interval. The total number of clutches initiated in each week is shown at the top of the bar. The last bar (labeled 20 June) collapses data for six clutches initiated over a period of 3 weeks ending July 11.

Sources of nest loss among moorhens included predation, desertion (or destruction) of the nest in response to parasitism or other disturbance. In the second half of the breeding season many nests on labile floating islands of vegetation were carried downstream with the river current. The owners abandoned these nests, and this represented an increasingly important source of nest loss for moorhens (Fig. 6). None of the moorhen nests on resident territories were lost this way. Resident moorhens, such as purple gallinules, nested in emergent vegetation or on solid substrate in sheltered bays without strong currents.

## Discussion

### CBP and life-history strategy in tropical rails

The population-wide rate of CBP for common moorhens in Panama (20% of nests) was within the range reported for temperate common moorhen populations (10–20% of nests [Britain], McRae 1995; up to 30% of nests [Japan], Ueda et al. 1993; 18% of nests [South Carolina, USA], Post and Seals 2000; 40% of nests [Netherlands], Petrie et al. 2009). Other territorial rails that show CBP include American coots (Weller 1971; Arnold 1987; Lyon 1993, 2003), soras *Porzana carolina* (Sorenson 1997), lesser moorhens *G. angulata* and African red-knobbed coots *Fulica cristata* (Jamieson et al. 2000), Argentinian red-fronted coots *F. rufifrons,* and red-gartered coots *F. armillata* (Lyon and Eadie 2004).

By contrast, there was no evidence of CBP by American purple gallinules. No previous reports of CBP in this species could be found, in spite of comprehensive research on its breeding behavior in Costa Rica (Hunter 1985, 1987), Louisiana, and Florida (Helm 1994). Nor has CBP been documented in well-studied populations of its Old World relative, the purple swamphen (*Porphyrio porphyrio*, Craig 1980; Jamieson et al. 1994). Ecological factors that reduce the opportunity for CBP in American purple gallinules in Panama include lack of host availability due to larger relative distances between conspecific territories, and year-round, asynchronous breeding. American purple gallinules showed more restrictive habitat use than tropical common moorhens preferring shallower river margins. Similar habitat partitioning was observed between these species in Florida (Helm 1994).

Tropical populations of birds commonly have reduced mean clutch sizes compared with temperate conspecifics (Stutchbury and Morton 2001). Mean clutch size of tropical common moorhens was not substantially lower than that of the European subspecies. Consistent with findings in a European population (McRae 1998), tropical common moorhens show remarkable among-female variation in clutch size. American purple gallinules had both a lower mean and a narrower range of clutch sizes than tropical moorhens. In species that regularly engage in CBP, individuals may adjust clutch size as part of a flexible life-history strategy (Lyon and Eadie 2008). Thus, differences in laying strategies (variation in clutch size and annual nesting rate) constitute major life-history trait differences between these species.

### Host responses to brood parasitism in tropical rails

Observations of natural CBP and experimental CBP in tropical common moorhens revealed that their responses vary in relation to the timing of parasitism, as has been shown previously in a European population (McRae 1995). However, tropical moorhens frequently rejected parasitic eggs laid during the host's laying period, mainly by ejecting them from the nest. Missing eggs could instead have been interpreted as instances of partial predation. Two lines of evidence suggest egg ejection is more likely. First, I never observed more than one egg disappear on the same day. In cases where real eggs were removed, it seems unlikely that a predator would fail to return to a known source of food. Second, both experimental and natural parasitic eggs were significantly more likely to disappear than host eggs or eggs from nonparasitized nests.

The rate of model egg rejection was lower that the rate of natural parasitic egg rejection. Experimental hosts may have had fewer cues upon which to base detection of parasitism. In European common moorhens, the host was more often than not on the nest at the time the brood parasite intruded (McRae 1996).

European moorhen hosts commonly deserted their nest if parasitized early in the host laying period, but only one European moorhen was observed to reject an experimental egg by burial (McRae 1995). In African lesser moorhens nesting in an ephemeral marsh with water less than a third of a meter deep (Jamieson et al. 2000), egg rejection in response to CBP was consistently by burial in the nest. Burial occurs as nest material is added, but hosts may risk damaging their own eggs as the nest material is manipulated under them. In shallow water, an egg ejected over the nest rim would draw the attention of visual predators. By contrast, the Rio Chagres is an extremely deep and fast-moving river. In this tropical river habitat, freshly laid eggs ejected out of floating nests sink beyond visibility. Thus, ejecting hosts achieve effective removal without attracting predators.

Egg rejection requires the ability to recognize foreign eggs, and should evolve only when the cost of parasitism is high. This is offset by the risk of mistakenly ejecting one's own egg (Davies et al. 1996; Davies 2000). There was some evidence that hosts in this population made occasional mistakes and ejected own eggs, but it is unclear whether this was due specifically to recognition errors. Hosts may have had difficulty removing eggs, or these host eggs may have disappeared by other means (e.g., been accidentally knocked out or broken). The use of a red experimental egg served to test whether hosts would reject when discrimination was made even easier. Though these experiments could not be completed in one season, the single assay resulted in egg rejection, as expected. Red eggs were commonly accepted in a study of European moorhens (McRae 1995). This does not indicate a perceptual constraint. Rather, it was argued that hosts in that population are not under strong selection to evolve rejection responses because (1) rates of parasitism were relatively low, and (2) parasitic eggs were rarely laid during the host laying period. Further, a cost-benefit model demonstrates that CBP early in the host laying period triggers host nest desertion, which is an effective antiparasite response under conditions of low CBP rates (McRae 1995). Further studies should compare populations in different geographic locations that are exposed to different levels of risk of CBP.

That American purple gallinules did not exhibit antiparasite defenses indicative of recognition supports that CBP is rare or lacking in this population. Documenting their behavior is nonetheless useful in revealing possible stages in the evolution of host responses (e.g., one individual deserted a Day 1 experimental egg and immediately resumed laying her clutch in another nest), and behavioral repertoires that could be exapted (e.g., a purple gallinule buried a model egg after the host eggs began to hatch). A parent that removes an egg that will not hatch (perhaps based on lack of vocalizations coming from the egg), may protect its brood from predators using visual or olfactory cues. Purple gallinules apparently “missed” laying an egg at 7 of 11 nests with good sequence data; this is probably explained by purple gallinules having an interegg interval of more than 24 hours.

### Ecological correlates of CBP

CBP in tropical common moorhens was restricted to nests in territories among the floating mats of vegetation that were temporary, and unpredictable once the rainy season begins. None of the nests in the sheltered bays occupied by year-round residents was parasitized. Earlier nest initiation dates and reduced risk of nest loss were additional advantages of remaining on these territories year-round. I cannot exclude the possibility that breeder age and experience influenced female moorhens’ reproductive decisions. However, moorhens on resident territories and on impermanent territories seemed to be using different strategies: only hens that defended seasonal territories in parts of the river where the currents put nests at risk of floating away engaged in CBP.

In contrast to wattled jacanas (*Jacana jacana*) that also occur at high density and nest year-round on floating islands of water lettuce in the same parts of the river (Emlen and Wrege 2004), tropical moorhens nesting on these ephemeral islands had a distinct breeding season. They nested over a period of 4 months, but peak laying occurred in late April (the end of the dry season). The majority of parasitic eggs were also laid in this period. Timing of breeding of common moorhens in the open water of the Rio Chagres was likely constrained by the accumulation of appropriate nesting vegetation. While a few resident pairs nested on the shore in bushes, hanging vines or even in an abandoned boat, the majority selected islands of floating hyacinth the availability of which increased over the course of the dry season. These islands provided good cover and reduced accessibility by terrestrial predators. Similar nest site selection in both emergent and floating mats of vegetation has been described for moorhens in North America (Greij 1994), and during the dry season on Guam (Takano and Haig 2004b). The occurrence of CBP in these populations has not been assessed.

Peak nesting in tropical moorhens coincided with the Rio Chagres reaching its lowest annual water level. However, the specific cues triggering synchronous breeding in tropical moorhens were not obvious. Accumulation of a critical mass of floating vegetation is one possibility. Day length may also be a contributing factor: tropical landbirds in Panama have been found to respond to the slightest changes in photoperiod (Hau et al. 1998). Additional years of study are needed to measure ecological correlates of nest timing and synchrony.

Tropical moorhens experienced a high rate of nest loss later when the water level rose. Nesting on hyacinth islands proved a liability when they floated downstream. Moorhens abandoned these nests, and this represented a major source of nest loss in the second half of the season. Thus, there was a strong effect of season on nest success: early clutches were significantly more likely to survive to hatch.

### The importance of risk in the evolution of CBP

Evidence from this and other studies of CBP in rails (McRae 1997; Jamieson et al. 2000) and waterfowl (Pöysä 1999) suggest a positive relationship between CBP rate and the rate of nest loss. In a year of exceptionally high nest predation, the proportion of eggs laid parasitically in a common moorhen population in Britain more than doubled. Predation on multiple nests within local areas increased synchrony of clutch initiation, and the frequency of parasitic eggs being laid in the host laying period (McRae 1997).

In a study of goldeneye ducks in Finland, brood parasitism rates among nest sites have been found to vary in relation to the risk of nest predation (Pöysä 1999). Incorporating data on spatial patterns of predation and parasitism into a clever model, Pöysä and Pesonen (2007) have demonstrated that risk assessment can be a vital predictor of rates of parasitism. Specifically, their model shows that females that adjust their laying tactics in relation to nonrandomly distributed nest failure rates are at a selective advantage. There is some experimental evidence for risk perception affecting individual laying behavior. Female goldeneye ducks cease laying parasitically in nest sites where they have experienced (simulated) nest predation, but the same sites are not avoided by females that did not themselves experience nest predation there (Pöysä et al. 2010). Further studies of CBP should attempt to investigate experimentally whether risk perception affects an individual's decision to lay parasitically.

That CBP by moorhens occurred on ephemeral territories and in nests on impermanent floating islands suggests that females in this tropical population may use this tactic primarily out of constraint (McRae 1998). Most studies of CBP in common moorhens have been conducted in managed areas with controlled water levels and predators (Gibbons 1986; McRae 1995; Post and Seals 2000; Forman and Brain 2004; Petrie et al. 2009). CBP rates may be higher where nesting birds experience higher predation rates and other natural hazards. Causes of nest loss other than predation include flooding and substrate instability. For example, in a study of wood ducks (*Aix sponsa*) using natural nest cavities, nest failure occurred due to nest site usurpation, cavity flooding, and floor collapse (Roy Nielsen et al. 2006a).

Tropical moorhen nests in relatively protected habitat near human habitation were not parasitized. Breeder age and experience likely influence whether or not females lay parasitically (see also McRae and Burke 1996; McRae 1998). It is conceivable that older birds were able to secure desirable territories in protected bays. Whether plasticity in reproductive tactics in other populations similarly correlates with habitat use or variation in wetland characteristics remains to be tested.

Studying the behavior and ecology of species, such as the common moorhen, whose distributions span a broad latitudinal range that expose them to diverse climatic conditions allows us to measure the effects of specific ecological variables and their relationships to life-history traits essential for predicting population dynamics. Moreover, the extent of phenotypic plasticity in reproductive behavior may foretell an animal's ability to cope with environmental challenges. Populations residing in the tropics experience more limited variation in climatic conditions and may be more vulnerable to environmental perturbations. Many tropical rails are island endemics. For example, the endangered Mariana moorhen (subspecies *G. c. guami*) suffers high nest loss due to invasive predators and negative impacts of invasive vegetation (Takano Haig 2004a). A dozen extinctions have occurred among tropical species of rail, including a moorhen endemic to Samoa, in the last 120 years (Taylor and van Perlo 1998). Future studies will need to address how ecological factors modified by climate change (seasonal variation in temperature, solar radiation, rainfall, humidity, etc.), pollution (e.g., oil spills), and other anthropogenic activities affect behavior and reproductive success, both at the level of the population and the individual.

## Supporting information

**Appendix S1.** Mean egg weights and dimensions (±SE) in the vicinity of Gamboa, Panama.
