# Supplementary material for: Conspecific brood parasitism in the tropics: an experimental investigation of host responses in common moorhens and American purple gallinules
Source: Ecol Evol. 2011 Nov;1(3):317–29. doi: 10.1002/ece3.26 (PMC3287317; doi:10.1002/ece3.26)
Supplement: Supplementary file 1 [file ece30001-0317-SD1.doc]

Appendix

Mean egg weights and dimensions (±SE) in the vicinity of Gamboa, Panama. Moorhen data were based on 55 females’ clutches of 4-10 eggs. Purple gallinule data were based on clutches of 3-5 eggs laid by 13 different females. All egg weights were measured within 24 hours of laying.

| Species | Mean weight | Weight range | Mean length | Range | Mean width | Range |
| --- | --- | --- | --- | --- | --- | --- |
|  | g | g | mm | mm | mm | mm |
| *Gallinula chloropus cachinnans* | 23.8 ± 0.3 | 19.0 – 28.1 | 43.9 ± 0.3 | 40.5 – 55.5 | 31.3 ± 0.2 | 29.0 – 37.8 |
| *Porphyrula martinica* | 17.5 ± 1.6 | 14.4 – 19.4 | 39.7 ± 1.6 | 35.6 – 42.0 | 28.4 ± 0.5 | 27.6 – 29.2 |
